# Antimicrobial adhesive films by plasma-enabled polymerisation of m-cresol

**DOI:** 10.1038/s41598-022-11400-8

**Published:** 2022-05-09

**Authors:** Hugo Hartl, Wenshao Li, Thomas Danny Michl, Raveendra Anangi, Robert Speight, Krasimir Vasilev, Kostya Ken Ostrikov, Jennifer MacLeod

**Affiliations:** 1 School of Chemistry and Physics and Centre for Materials Science, Queensland University of Technology (QUT), 2 George Street, Brisbane, QLD 4000 Australia; 2 School of Biology and Environmental Science and Centre for Agriculture and the Bioeconomy, Queensland University of Technology (QUT), 2 George Street, Brisbane, QLD 4000 Australia; 3 UniSA STEM, University of South Australia, Mawson Lakes, SA 5095 Australia; 4 Fachhochschule Nordwestschweiz (FHNW), Hochschule Für Technik, Institut Für Nanotechnische Kunststoffanwendungen, Klosterzelgstrasse 2, 5210 Windisch, Switzerland; 5 ARC Centre of Excellence in Synthetic Biology, Queensland University of Technology (QUT), 2 George Street, Brisbane, QLD 4000 Australia; 6 College of Medicine and Public Health, Flinders University, Bedford Park, SA 5042 Australia

**Keywords:** Surfaces, interfaces and thin films, Design, synthesis and processing

## Abstract

This work reveals a versatile new method to produce films with antimicrobial properties that can also bond materials together with robust tensile adhesive strength. Specifically, we demonstrate the formation of coatings by using a dielectric barrier discharge (DBD) plasma to convert a liquid small-molecule precursor, m-cresol, to a solid film via plasma-assisted on-surface polymerisation. The films are quite appealing from a sustainability perspective: they are produced using a low-energy process and from a molecule produced in abundance as a by-product of coal tar processing. This process consumes only 1.5 Wh of electricity to create a 1 cm^2^ film, which is much lower than other methods commonly used for film deposition, such as chemical vapour deposition (CVD). Plasma treatments were performed in plain air without the need for any carrier or precursor gas, with a variety of exposure durations. By varying the plasma parameters, it is possible to modify both the adhesive property of the film, which is at a maximum at a 1 min plasma exposure, and the antimicrobial property of the film against *Escherichia coli*, which is at a maximum at a 30 s exposure.

## Introduction

In recent years, the impetus to create coatings on-surface with antimicrobial properties has increased^1^. Although many antimicrobial coatings target applications in healthcare settings and consumer products^2,3^, potential applications are far-reaching, including use in maritime settings^4,5^, and building exteriors^6–8^. Producing these large-area coatings from waste products, such as those created through the refinement of coal to coal tar, represents an interesting opportunity to simultaneously minimise costs and value-add to a high-abundance, low-cost precursor. Coal tar is a by-product produced during the synthesis of coke and coal gas from coal^9^. Coal tar has been used to create useful materials, such as anode materials for lithium-ion batteries^10^, supercapacitor electrodes^11^, optically polarising films^12^, and carbon nanosheets^13,14^. The process of synthesising oil from coal on large scales results in a constant supply of coal tar, along with its concomitant by-products including cresols^15,16^. The global production was 137.5 kilotons in 2015^17^ and is continuing to rise as cresols can be used as precursors to numerous polymers, pharmaceuticals, pesticides and dyes^18^. Cresols have inherent disinfectant properties^19–21^, and are naturally found in coal tar, resin, pitch and crude oil^20,22^. Here we focus on m-cresol, as it is the only form of cresol that is liquid at room temperature. Furthermore, m-cresol is the highest constituent in the phenol oil extracted from tar^23^. m-Cresol is used by manufacturers as a preservative in antivenoms^24^, as a general antimicrobial preservative^25,26^, and as a disinfectant^27^.

As we try to move towards a waste-free world, considerable effort and creativity have been dedicated to the transformation of waste products into useful films, such as anti-corrosion films formed from powdered egg shells on stainless steel^28^, UV-protective films made from hemp fibres and polyvinyl alcohol^29^, antioxidant and pH indication from chitosan and food waste^30^, and water barriers from biomass^31^. The incorporation of antimicrobial agents created from waste products into films such as polylactic acid or polyethylene has been reported^32,33^, however, descriptions of the direct synthesis of antimicrobial coatings solely from waste products is much more limited. Coatings with antimicrobial properties have been created by incorporating inorganic active agents such as silver nanoparticles^34^, molecules that activate upon contact such as peptides^35^, antibacterial agents such as antibiotics^36^, organic agents such as chitosan^37,38^, and materials that impact the bacterial membrane such as graphene^39^, chloramine^40^, or potassium sorbate^41,42^. Fabricating antimicrobial coatings on-surface can be time-consuming and expensive^43–45^, and the practical manufacturing of these coatings on large scales and useful surfaces is limited, with coating synthesis generally slowed and complicated by long multi-step processes^43–47^. Cheap and fast processes are required to coat large areas such as buildings and maritime structures.

In order to provide durable and long-lasting protection, an antimicrobial coating must be strongly adhered to its support surface. One approach to this challenge is to utilise an adhesive antibacterial surface^48^. Adhesives stick due to their molecular bonds to the surface, and the strength needed to break these bonds dictates the strength of the adhesives. Synthetic adhesives have been created by materials such as hydrogels^49^, methacrylate^50,51^, polyethylene^52^, phloroglucinol^53^, and chitosan-glycerin^54^. However, the synthesis of synthetic polymer glues is often complicated by high costs^55^, high cure temperatures and long cure times^55^, and they require the mixing of multiple parts to cure them^56^.

**Figure 1:**
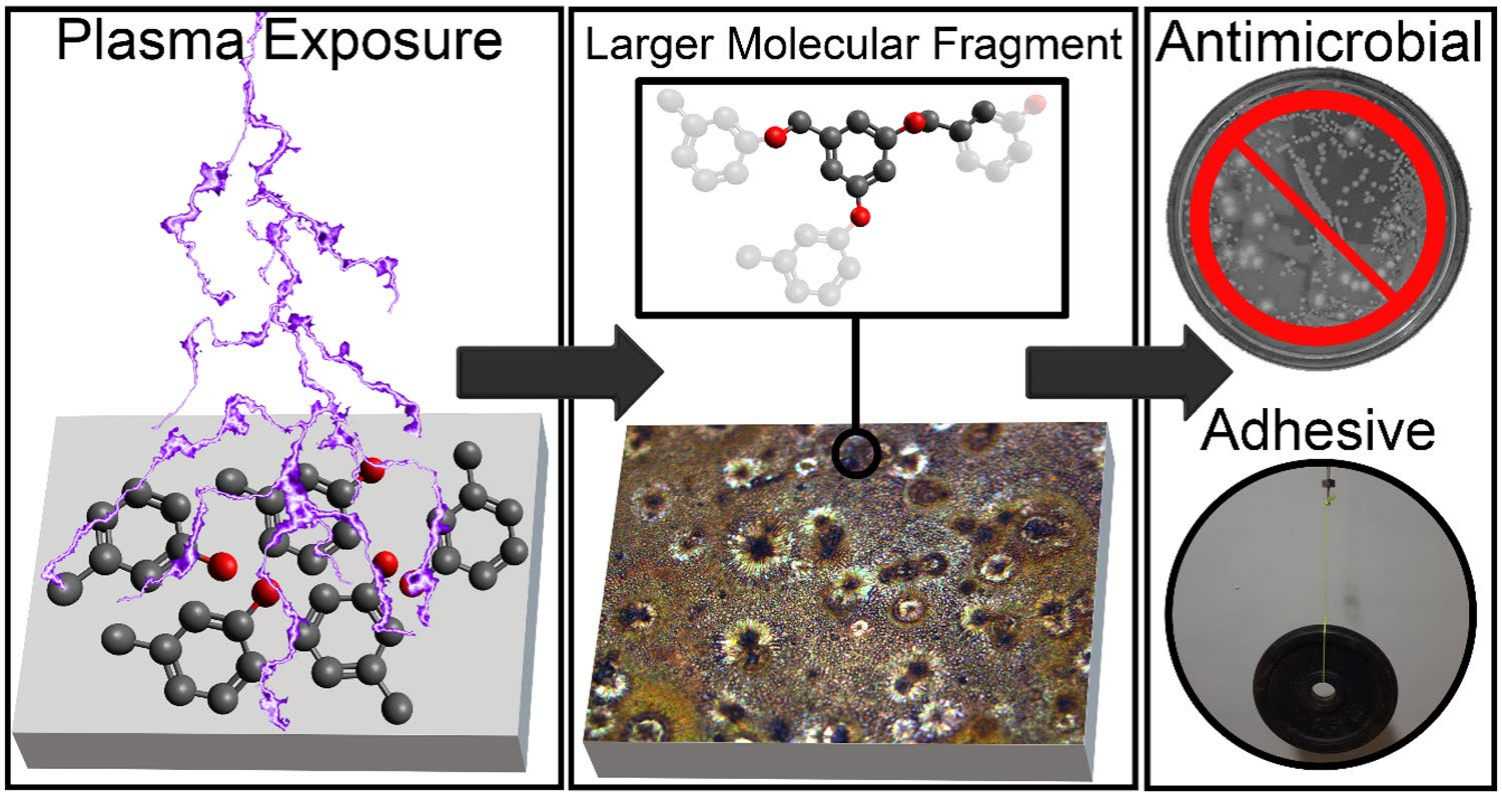
The concept of the polymerisation of m-cresol by plasma exposure on a substrate, creating coatings with antimicrobial properties and notable tensile bond strength as an adhesive, gluing substrates together strongly enough to support a hanging weight of 5 kg. Shown in the figure is a possible interpretation of the molecular fragment C_10_H_5_O_3_- identified by ToF–SIMS, and a proposed oligomer consistent with this fragment. For this proposed fragment, we hypothesise that m-cresol monomers link through C–O–C bonding.

Plasma polymerisation for the synthesis of coatings is a well-established area of research, with the majority of research focussing on gas-phase precursors fed into a plasma, or by ignition of a plasma within a liquid precursor solution^57–59^. In this research we instead investigate a relatively unexplored liquid-to-film approach, wherein a droplet of small molecule liquid precursor is reacted through a plasma contained to a chamber, producing a coating directly on the substrate. The polymerisation of small molecules by plasma is an emerging area of research, where biofilm-resistant coatings have been produced from (2,2,6,6-tetramethylpiperidin-1-yl)oxyl (TEMPO)^60^, coatings that limit bacteria attachment have been synthesised from terpinen-4-ol^61^, and antibacterial coatings from 1,8-cineole^62^. Here, we demonstrate a versatile approach to synthesising films with antimicrobial properties that form a bond with high tensile adhesive strength, and can be formed on many surfaces (see Fig. 1). In this work, we use an atmospheric-pressure, room-temperature plasma, which allows for greater cost-efficiency, and a simpler and quicker setup as compared to low-pressure plasma systems^63^. The process involves deposition of a small molecule liquid organic precursor onto a substrate, which is then exposed to a dielectric barrier discharge (DBD) plasma. Within the reactive environment of the DBD plasma, the precursor will be exposed to electrons, ions, ultraviolet radiation, free radicals, and other excited species, which can all contribute to reactions in suitable precursor molecules. We have previously shown that a plasma could be used to cleave carbon-halogen bonds in other small molecules to form a solid film on-surface. However, that substrate-catalysed process was limited to occur only on metal substrates^64,65^. In this work, we present a polymerisation process that requires no metal catalyst, allowing the application of the coatings to a much wider selection of substrates, and thus would be simple to scale to larger coatings, for example by using roll-to-roll plasma processing^66^. In this new method, we hypothesise that we polymerise the m-cresol precursor by dehydroxylation and C–H bond breaking, with subsequent C–C coupling, leading to a liquid–solid transition. This method of plasma polymerisation is particularly promising for scale-up, as it can be performed at room temperature and pressure.

## Results

### Film morphology

**Figure 2:**
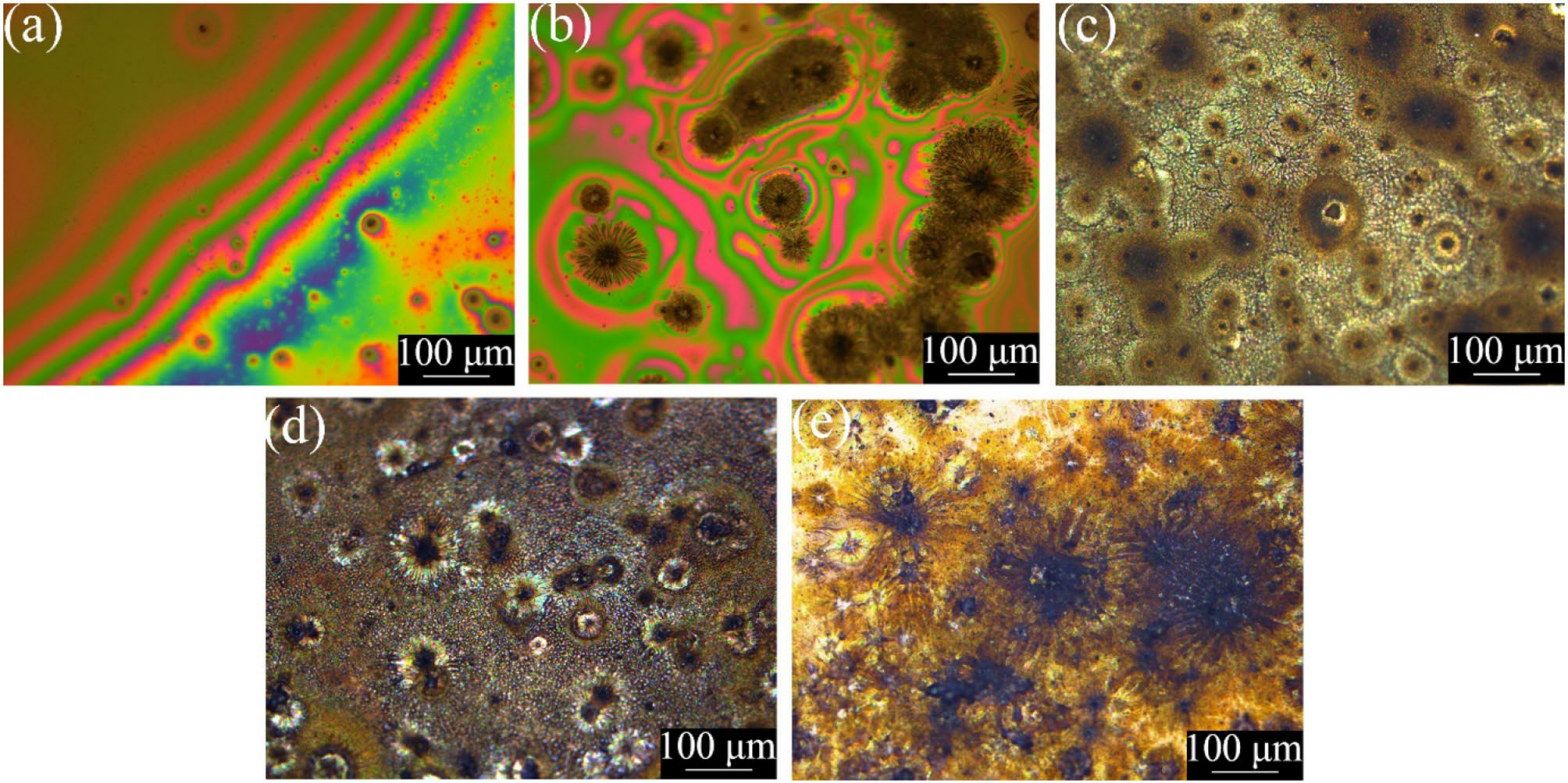
Optical images displaying the features of the films of m-cresol created by plasma exposure durations of (**a**) 0.5 min, (**b**) 1 min, (**c**) 2 min, (**d**) 3 min, and (**e**) 4 min on a Si substrate.

**Figure 3:**
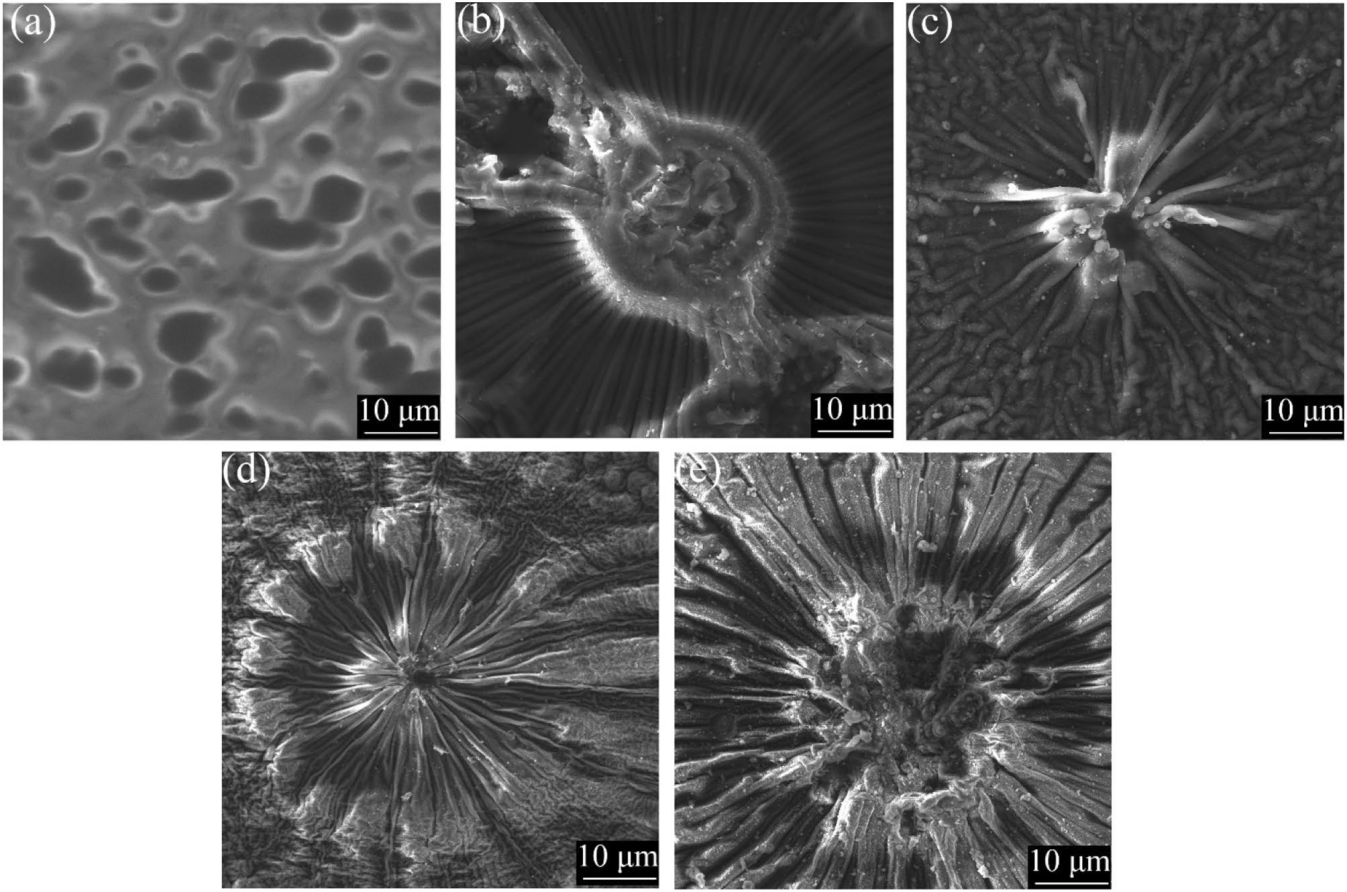
SEM images displaying the features of the films of m-Cresol created by plasma exposure durations of (**a**) 0.5 min, (**b**) 1 min, (**c**) 2 min, (**d**) 3 min, and (**e**) 4 min, on the Si substrate.

For all plasma exposures we observed a liquid-to-solid transition of the m-cresol during plasma processing, suggesting that higher molecular weight products were produced. The colouration and morphology of the films differed with plasma dose (power × time) (see Fig. 2). The films appear to be initially smooth, until eventually sunburst features appear, increasing in number with plasma exposure time. Thin film interference effects are seen in the first two samples. Scanning electron microscopy (SEM) imaging of the films reveals similar features to optical microscopy (see Fig. 3). No visible damage was seen to occur to the films during SEM imaging, and the films did not appear to charge when exposed to the electron beam.

### Film chemistry

**Figure 4:**
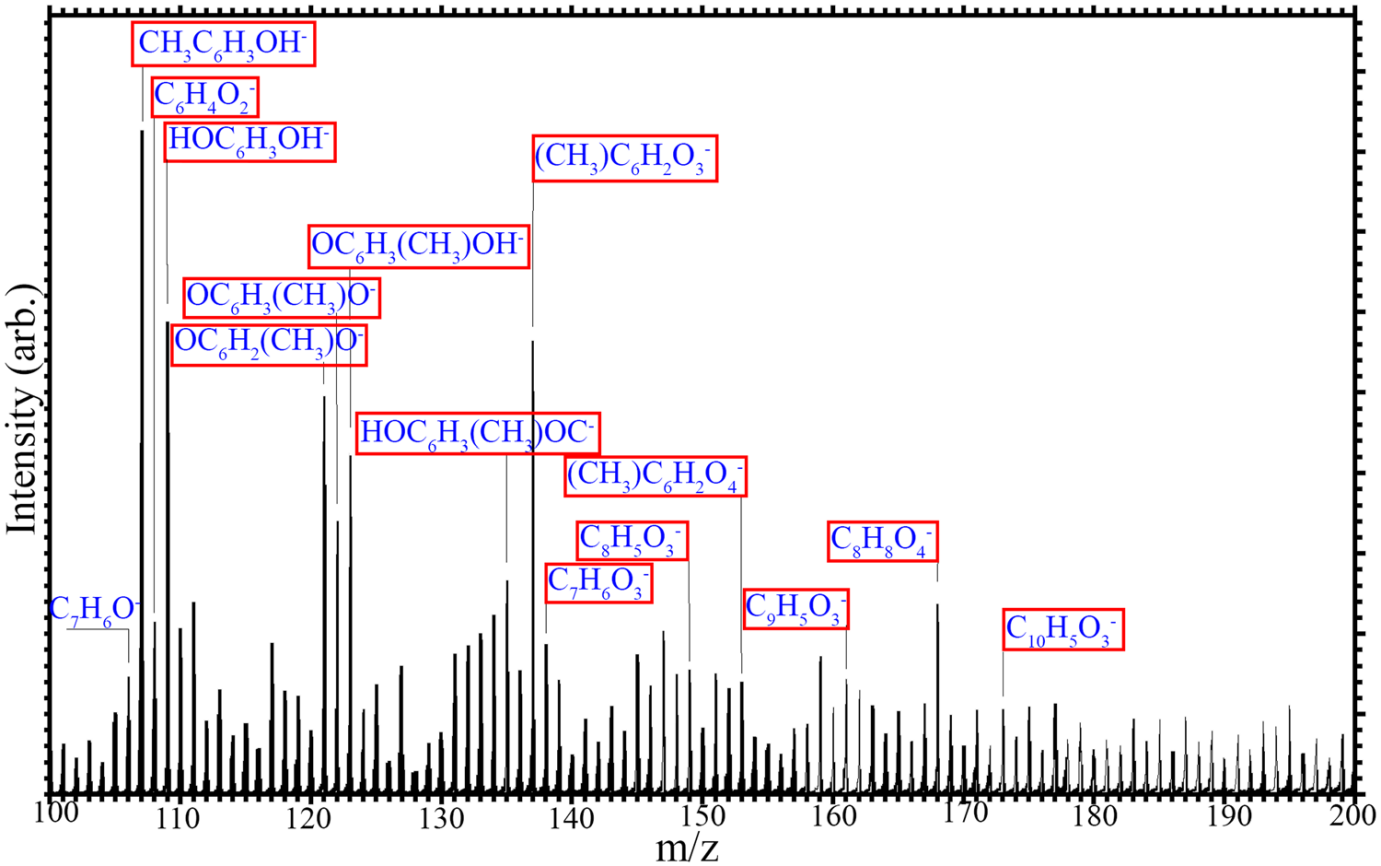
The ToF–SIMS results with negative polarity of an m-cresol film produced with a 1 min plasma exposure, with fragments highlighted in red that suggest oligomerisation or polymerisation of m-cresol.

The chemistry of the film produced by a 90 W plasma exposure for 1 min was investigated with ToF–SIMS, which can help identify products through their fragmentation under ion sputtering. ToF–SIMS shows a product containing fragments that suggest multiple bonded benzene rings such as C_9_H_7_ (115.15 m/z), and C_8_H (97.10 m/z), including structures with attached hydroxyl groups such as C_7_H_7_O_2_ (123.13 m/z), C_10_H_5_O_3_ (173.15 m/z), C_8_H_8_O_4_ (168.15 m/z), and C_9_H_5_O_3_ (161.14 m/z), suggesting oligomerisation or polymerisation has occurred (see Fig. 4, Figs. A.6 and A.7). Nitrogen, a component of the air atmosphere that the plasma was produced in, is also seen bonded to the product fragments, such as C_6_H_8_N, C_5_H_6_N, C_3_H_8_N, and C_2_H_6_N, which are similar to other fragments we have identified before in other polymers synthesised through DBD plasma polymerisation under nitrogen atmosphere^65^. Silicon from the substrate is not bonded in any fragments. Small levels of contaminants in the form of Na and Fe are also seen, expected to be contamination during the plasma process. Interestingly, modified forms of m-cresol are seen as fragments, such as benzene-like fragments at 77.11 m/z (m-cresol with both functional groups cleaved), and toluene-like fragments at 91.13 m/z (m-cresol with just the OH group cleaved).

### Tensile testing

**Figure 5:**
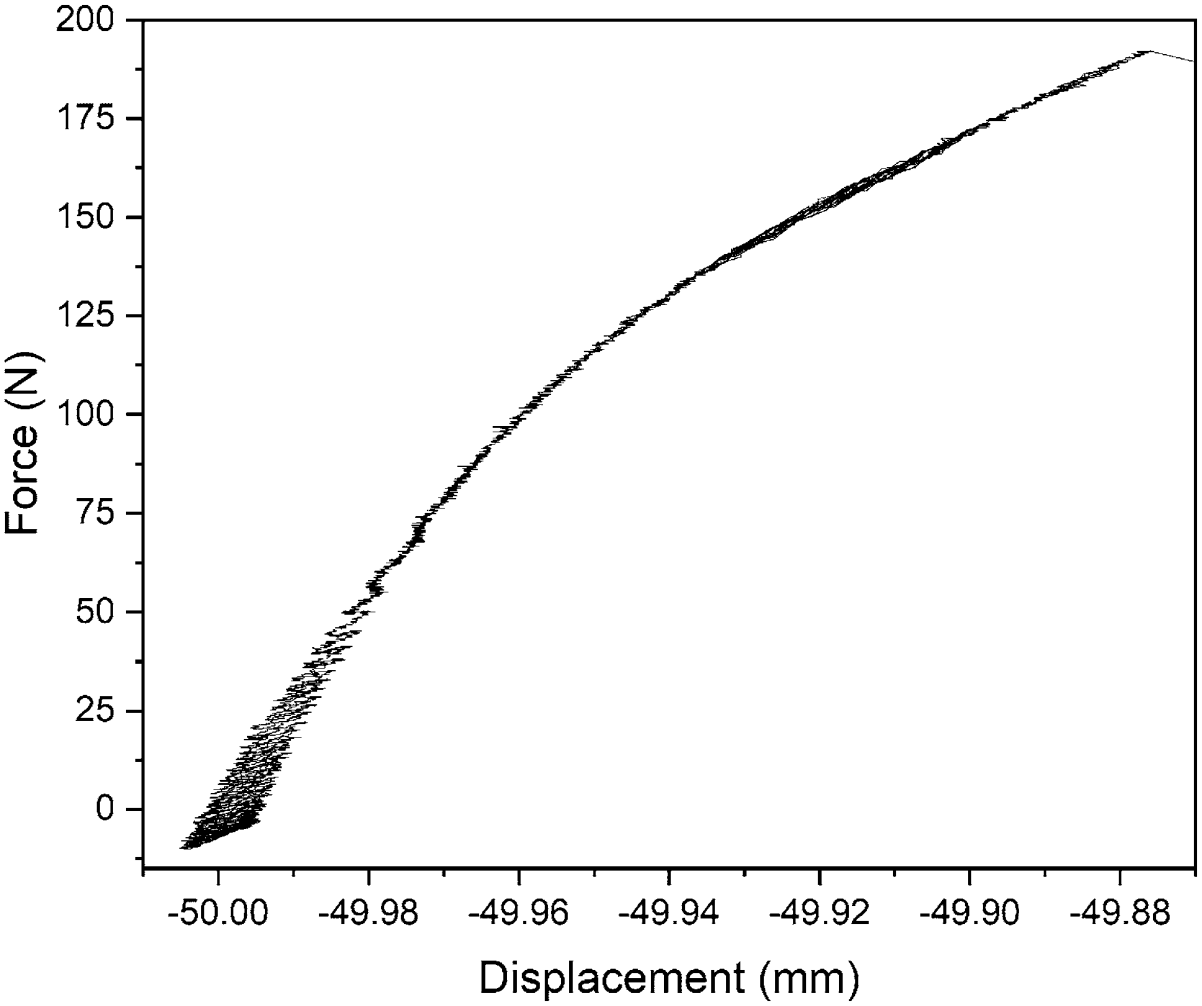
Tensile testing of the m-cresol bond, showing a force of ~ 192 N required to break the bond, with the film stretching a length of ~ 120 µm.

Tensile testing (see Fig. 5) was performed on a steel assembly bonded using an adhesive m-cresol-derived film (see Fig. A.2). The tensile strength of the m-cresol film was ~ 192 N/cm^2^ and the film stretched ~ 120 µm before breaking adhesion. An image of the film after the adhesive bond was broken can be seen in Fig. A.3.

### Antimicrobial testing

**Figure 6:**
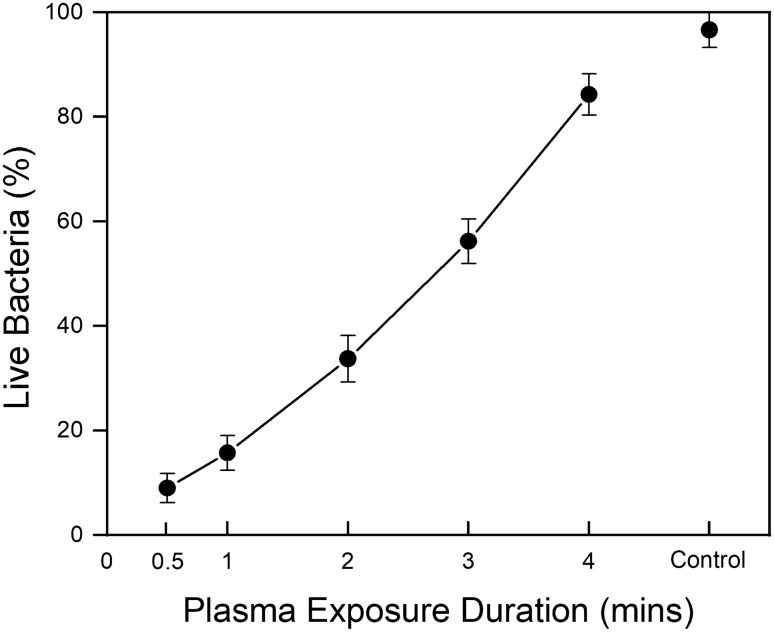
Percentage of live *E. coli* observed after 15 min contact on cresol-derived films synthesised using different plasma exposures, compared to the uncoated silicon wafer control. A line between each data point guides the eye for the antimicrobial activity of the films, and error bars show the standard deviation of live bacteria, calculated from triplicate tests of the dot point mean values. A similar level of uncertainty is seen in the coated samples as in the control, and we attribute this to the uncertainty produced from the expected slightly variable natural death rate of the triplicate bacteria solutions.

m-cresol naturally has antimicrobial properties, and thus one could reasonably expect its polymerised product may share this attribute. Five m-cresol films were created using varying plasma exposures, and were then washed and heated before being tested for their antimicrobial properties. We also tested a silicon wafer with no film as a control. The results show that the lowest plasma dose of 0.5 min produces a film with a very high level of antimicrobial activity (See Fig. 6). The antimicrobial activity then drops with increasing dose duration, eventually reaching approximately control levels at 4 min of plasma exposure.

## Discussion

DBD plasma exposure facilitates the creation of solid thin films from an m-cresol precursor. Testing on various substrates shows that DBD plasma can be used to polymerise m-cresol on substrates such as glass, Teflon, Si and steel (see Figs. A.9 and A.10). At the lowest plasma exposures, the films appear smooth, whereas SEM imaging reveals that in films exposed to higher plasma doses, sunburst-type features appear as a dominant motif. We have observed similar features in our investigations of the DBD polymerisation of other small molecules^64^. We propose that these might be Lichtenberg figures that have formed due to the electrical current passing through the material^67^.

Polymerisation of m-cresol is expected to occur by a combination of a dehydroxylation reaction (where the hydroxyl group is cleaved), and breaking of C–H bonds, with the activated molecules then linking via C–C coupling. ToF–SIMS showed a product that contains multiple bonded benzene rings with attached hydroxyl groups, which confirms that oligomerisation occurs, and suggests that C–H bond breaking may be the dominant reaction. Nitrogen is also seen as a component of the product created, whilst the silicon substrate does not appear to play any part chemically in the film makeup at the surface, as we do not see this bonded in any fragments. Fragments smaller than m-cresol, such as benzene and toluene, are also observed, suggesting that one or more functional groups are removed from m-cresol prior to its oligomerisation. The colouration of the film surface appears to darken with increasing plasma dose, suggesting further conversion to amorphous carbon. The dark films do not transmit ultraviolet light (see Fig. A.8).

At a total plasma exposure duration of one minute, the chemistry of the film is sufficient to produce a high tensile-strength bond. These coatings were found to have an adhesion value of ~ 192 N/cm^2^. To compare, two-part epoxies have a strength of approximately 200–600 N/cm^2^, and cyanoacrylate-based super glues have typical strengths of 600–2000 N/cm^2^^68,69^. The synthesis of synthetic polymer glues (polymers that do not naturally occur in nature) is often complicated by high costs^55^, high cure temperatures and long cure times^55^, and they require the mixing of multiple components for curing^56^. The tensile strength of these adhesives is often similar to that witnessed here, where we require only a cheap waste-product precursor, and a simple process of two plasma exposures. The films adhere well to a number of different substrates when tested by scotch tape lift-off (see Fig. A.13).

In tests of the antimicrobial properties of these films using *E. coli*, the sample formed with the lowest plasma dose showed the highest antimicrobial properties. The lowest dose sample (0.5 min plasma exposure) corresponded to a cell death of ~ 91%. Antimicrobial activity inversely correlates with the plasma dose used to form the films, until it almost reaches zero antimicrobial activity on a film formed from four minutes of plasma exposure. Additional testing where the film was submerged inside the microbial liquid, instead of applying the *E. coli* through a droplet on the surface, shows a similar trend (see Figs. A.4 and A.5). m-Cresol has been shown to be toxic to the growth process of bacteria, without any effect on the average cell size^70^. The antimicrobial effect of m-cresol is thought to be caused by physical damage to bacterial cell membranes^21^. This is proposed to be due to m-cresol binding or contacting with, and causing changes to the permeability of the osmotic barrier of a bacteria cell membrane, which causes the uncoupling and leaking of cytoplasmic constituents^21^. The chemistry of the molecule is also important, with research having shown that the combination of a benzene ring with a hydroxyl functional group is important to produce antibacterial activity^71^. m-Cresol has also been shown to cause protein aggregation (the clumping of mis-folded proteins) by partial protein unfolding^25^, an effect that correlates with the hydrophobicity of a molecule^72^. We therefore assume that both the polar hydroxyl and the hydrophobic (nonpolar) methyl functional groups may contribute to the antimicrobial activity initially in the unreacted molecule, and both groups are consistent with fragments of the final product as probed by ToF–SIMS. With increased plasma exposure, we anticipate that we may be modifying the chemical structure of the m-cresol significantly enough to compromise the antimicrobial properties. In general, the synthesis of antimicrobial surfaces can be time-consuming, complicated, expensive, and not suitable for scale-up^43–47^. Here, we improve on these points by only requiring a cheap waste-product precursor, and a fast process involving a single plasma exposure.

To ensure that retained, unreacted m-cresol was not responsible for the observed antimicrobial properties, we compared as-prepared films with films that underwent washing and heating to remove any retained precursor. The washed and heated films do not have significantly lower antimicrobial activity than the as-prepared films (see Figs. A.4 and A.5), suggesting that this property arises from the polymerised product itself, and not from unreacted m-cresol present. Leachable testing on washed and heated films shows that, within detection limits, we do not detect any m-cresol or larger oligomers in a 10 µL droplet of distilled deionised water left on the film for 15 min (see Figs. A.14–A.18). We did observe that the as-prepared films are more hydrophilic than those that had been washed and heated (see Table A.2). This change in wettability does not appear to have much effect on the antimicrobial properties, despite its implications for differing levels of contact of the microbial liquid with the surfaces.

Finally, we highlight how cost efficient this plasma-based process is. Operating the 90 W plasma for one minute consumes only 1.5 Wh of electricity to create a 1 cm^2^ film. Assuming linearity in scale-up, this process could produce 1 m^2^ of product using only 15 kWh of power, at a typical cost of $1.95 USD (taking an electricity price of $0.13 USD/kWh). The precursor is a cheap waste-product that can be purchased in bulk. There is no carrier or precursor gas required, meaning that the plasma exposure can be performed in open-air for even greater cost efficiency and simplicity. The substrate is also used as received, requiring no energy use to prepare, therefore increasing the cost-efficiency of the process. This process is therefore amenable to scale up. Film growth could, for example, be performed on a larger scale by spraying the liquid m-cresol precursor onto substrates, before passing them under several plasma jets on an industrial manufacturing platform, and would be expected to maintain the same or even greater energy efficiency^66^.

## Conclusion

We have demonstrated a new route to producing antimicrobial thin films with high tensile adhesive strength. These well-adhered films could be well-suited to applications in demanding environments such as building exteriors, or in maritime environments. We synthesise these coatings on-surface by using room-temperature atmospheric-pressure DBD plasma to polymerise m-cresol, a cheap waste-product. This occurs in an air atmosphere, without the requirement for any catalyst.

We hypothesise that the polymerisation of m-cresol proceeds by dehydroxylation and/or C–H bond breaking, followed by C–C coupling. The plasma parameters have a direct effect on the level of antimicrobial ability of the film, and of the tensile strength of the bond that the film can create between two surfaces. The antimicrobial properties are at maximum in the film produced at a 30 s plasma exposure, with an *E. coli* cell death of ~ 91% after 15 min. At a total exposure of 1 min, the tensile adhesive strength is ~ 192 N/cm^2^, approaching that of certain epoxy glues. These two functional properties are both significantly strong at a plasma exposure of just 1 min, meaning that these properties could be exploited together in applications such as maritime use.

This work therefore demonstrates a proof-of-principle of on-surface synthesis of antimicrobial coatings with high tensile bond strength through a new plasma-assisted liquid–solid transition reaction. This simple, quick process could translate into a technology for producing films with interesting properties on large scales. In the next steps we aim to upscale these coatings to evaluate how their physical and chemical properties translate onto larger surfaces.

## Methods

m-Cresol (99%, Sigma Aldrich) was selected as the precursor due to its liquidity at room temperature. Plasma treatments were performed using a dielectric barrier discharge (DBD) apparatus at room temperature (see Fig. A.1). Samples were placed on a quartz plate with a high-voltage AC generator (CTP-2000 K, Corona Laboratory). The plasma treatments were performed in open air (no sheath gas was used), for maximum cost-efficiency coatings. During sample preparation, 10 µL of m-cresol was pipetted onto stationary samples of steel and silicon. Samples were then exposed with plasma immediately following precursor deposition. The distance between the top of the DBD chamber and the silicon substrate was ~ 7 mm, whereas this spacing was ~ 6 mm for the steel substrate. The peak–peak output voltage of the plasma was ~ 30 kV at 40 kHz (measured by a Rigol DS6104 digital oscilloscope) for all samples, with the output power and current of the plasma increasing linearly with increasing input power. The high voltage output was achieved using a 0–250 V input voltage regulator. The waveform and power output of the plasma produced are consistent with those reported by others using the same equipment^73^.

Scanning electron microscopy (SEM, TESCAN MIRA3) and optical microscopy (Leica DM6000M) were used to characterise the morphology of the product on the surface. An accelerating voltage of 10 kV and a beam current of ~ 100 pA was used for SEM imaging, with samples analysed uncoated. A 20 × objective was used during optical microscopy. ToF–SIMS data were acquired using an IONTOF M6 instrument (ToF–SIMS, IONTOF GmbH) equipped with a reflectron time-of-flight analyser and Bi/Mn primary-ion source. Bi_3_^+^ cluster ions were selected from the pulsed primary-ion beam for the analysis and bunched to minimise the pulse length, thereby maximising the mass resolution (Δm/m = 7000 for C_2_H_3_^+^ and C_2_^−^). The primary-ion dose was limited to 10^11^/cm^2^, which is below the static limit. Data were acquired from 500 µm × 500 µm regions of the sample whilst flooding with low-energy electrons (21 eV) to compensate for surface charging. A cycle time of 140 µs provided an accessible mass range of m/z 0–1000 for the secondary ions. Data were acquired in both positive and negative polarity, and the mass scales calibrated using peaks attributed to hydrocarbon ions (C^+^, CH^+^, CH_2_^+^, C_2_H_3_^+^, C_3_H_5_^+^, C_(4–7)_H_7_^+^; C^−^, CH^−^, CH_2_^−^, C_(2–4, 6, 7)_^−^). The pressure in the analysis chamber during data acquisition was at, or below, 3 × 10^−9^ mbar.

For the tensile testing, two stainless steel (a common material structurally bonded by adhesives in aerospace use^74^) 10 mm × 10 mm squares with tapped M4 holes, were placed in the DBD chamber next to each other. 10 µL of m-cresol was pipetted onto each piece, and then they were exposed to the plasma for 0.5 min together. After this, both pieces were pressed together, such that the two film-coated faces were touching. The assembly was then exposed to a further 0.5 min of plasma. After this, threaded rods were screwed into the tapped holes, ready for tensile testing (see Fig. A.2) with Microforce testing (MTS Tytron Microforce Tester). The tests were performed in triplicate.

For the antimicrobial testing, 10 µL of m-cresol was pipetted onto 10 mm × 10 mm Si wafers (Ted Pella, Inc.), and five samples were made at varying exposures (see Table A.1). Samples were then heated to 210 °C (above the boiling point of m-cresol) for 5 min, and washed with distilled water (in which m-cresol is soluble) to remove any possible unreacted m-cresol from the surface. Microbial toxicity assays were then performed on all coated samples, as well as an uncoated silicon wafer as a control (an unmodified wafer, Ted Pella, Inc.). Cell viability was assessed by applying hemocytometer with added trypan blue solution, at 0.4% by volume (Gibco™, Thermo Fisher Scientific, AU), using the dye exclusion test. The trypan blue exclusion test is based upon the concept that live cells do not absorb trypan blue or other impermeable dyes, while dead cells are permeable and will readily take up the dye, allowing counting of live and dead cells^75^. *Escherichia coli* (*E. coli)* K12 ER2738 (QUT stock collection) was selected and harvested in LB medium at 37 ℃ and shaken overnight at 250 rpm until the OD_600_ reached 0.4. The cell culture was then diluted by 1:10. 10 µL of diluted cell culture was added onto each m-cresol coated sample, and was left stationary for 15 min. The culture from the surface of the coated samples was then transferred into 1.5 mL tubes by pipette and mixed with 10 µL of trypan blue solution. Next, 10 µL of mixed cell culture was added into cell counting chamber slides (0.1 mm, OPTIK-Labor, DE). The cells were then observed under a light microscope (Axioscope 5, ZEISS, AU) with the supporting software Labscope v3.1 (ZEISS, AU). Live and dead cells were counted and recorded. The test was performed in triplicate, and the mean and standard deviation of the results calculated to create error values.

Films were also analysed without any washing and heating (see Table A.1 for plasma parameters). Films without washing or heating appear similar by optical microscopy to those that were washed and heated, with films formed at higher doses appearing inhomogeneous (see Fig. A.11). The same sunburst features are also seen by SEM in the films without washing or heating, with higher dose films appearing inhomogeneous (see Fig. A.12).

## Supplementary Information

Supplementary Information.

## Data Availability

Supplementary information accompanies this paper at http://www.nature.com/scientificreports.
