# Supplementary material for: Antimicrobial adhesive films by plasma-enabled polymerisation of m-cresol
Source: Sci Rep. 2022 May 9;12:7560. doi: 10.1038/s41598-022-11400-8 (PMC9085887; doi:10.1038/s41598-022-11400-8)
Supplement: Supplementary file 1 — Supplementary Information. [file 41598_2022_11400_MOESM1_ESM.docx]

**Electronic Supplementary Information**

**for**

**Antimicrobial Adhesive Films by Plasma-Enabled
Polymerisation of m-Cresol**

Hugo Hartl^1^, Wenshao Li^2^, Thomas Danny Michl^3,4^, Raveendra Anangi^2^, Robert Speight^2,5^, Krasimir Vasilev^6^, Kostya (Ken) Ostrikov^1^, and Jennifer MacLeod^1,*^

^1^ School of Chemistry and Physics and Centre for Materials Science, Queensland University of Technology (QUT), 2 George Street, Brisbane, QLD 4000, Australia.

^2^ School of Biology and Environmental Science and Centre for Agriculture and the Bioeconomy, Queensland University of Technology (QUT), 2 George Street, Brisbane, QLD 4000, Australia.

^3^ Future Industries Institute, University of South Australia, Mawson Lakes, SA 5095, Australia.

^4^ Fachhochschule Nordwestschweiz (FHNW), Hochschule für Technik, Institut für Nanotechnische Kunststoffanwendungen, Klosterzelgstrasse 2, Windisch 5210, Switzerland.

^5^ ARC Centre of Excellence in Synthetic Biology, Queensland University of Technology (QUT), 2 George Street, Brisbane, QLD 4000, Australia.

^6^ College of Medicine and Public Health, Flinders University, Bedford Park, SA 5042, Australia. ^*^jennifer.macleod@qut.edu.au

Contents

[Plasma Setup and Parameters 2](#_Toc93954973)

[Tensile Testing 3](#_Toc93954974)

[Antimicrobial Testing of Films by Submersion with and without Washing or Heating 3](#_Toc93954975)

[ToF-SIMS 6](#_Toc93954976)

[Spectrometry 7](#_Toc93954977)

[Water Contact Angle 8](#_Toc93954978)

[Films Made on Other Surfaces 9](#_Toc93954979)

[SEM and Optical Microscopy of Films without Washing or Heating 10](#_Toc93954980)

[Adhesion Testing 11](#_Toc93954981)

[Leachable Testing 12](#_Toc93954982)

# Plasma Setup and Parameters

**
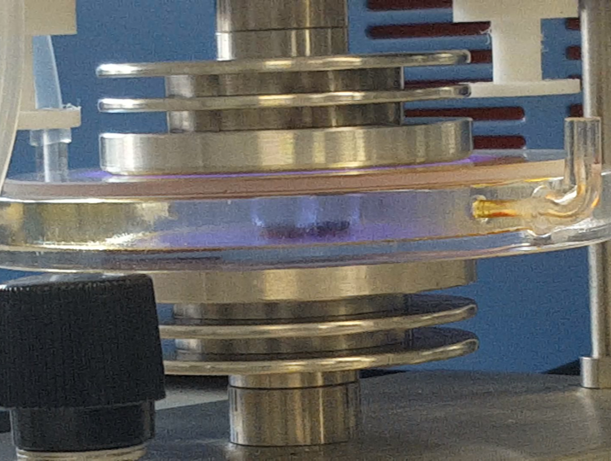
**

**Figure A.1.** The plasma reactor setup.

**Table A.1.**The plasma parameters of m-cresol exposures used for the various characterisations, all at a power level of 90 W.

| Tensile Testing (mins) | Antimicrobial Testing (Washed+Heated) (mins) | Antimicrobial Testing (mins) | ToF-SIMS, spectrometry, leachable testing  (mins) |
| --- | --- | --- | --- |
| 0.5+0.5 | 0.5, 1, 2, 3, 4 | 0.5, 1, 2, 3, 4, 5 | 1 |

# Tensile Testing


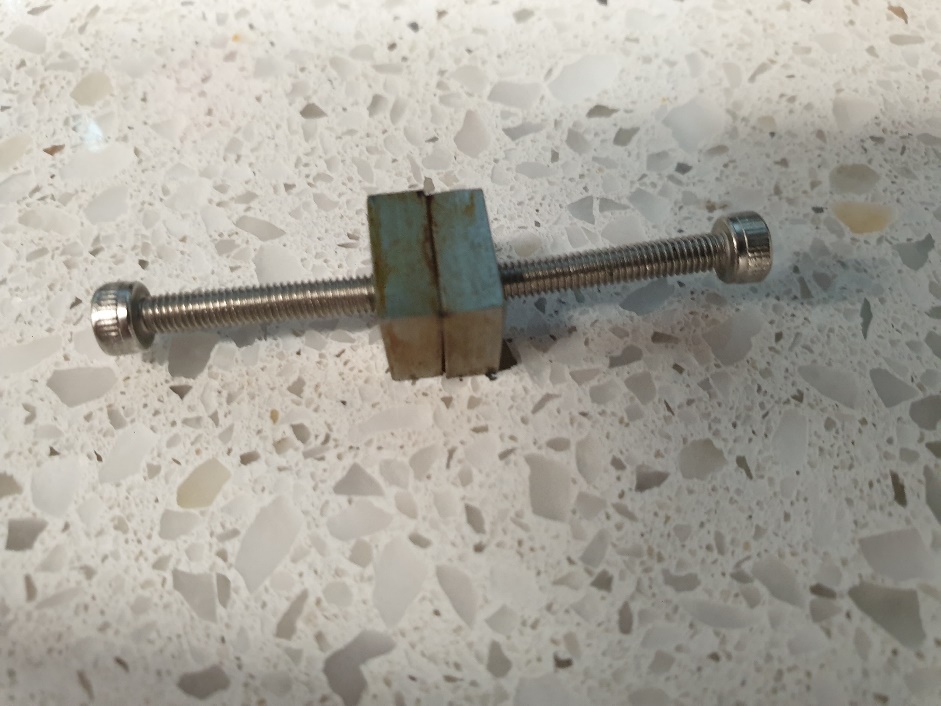


**Figure A.2.** The two films stuck together, ready for tensile testing of the bond.


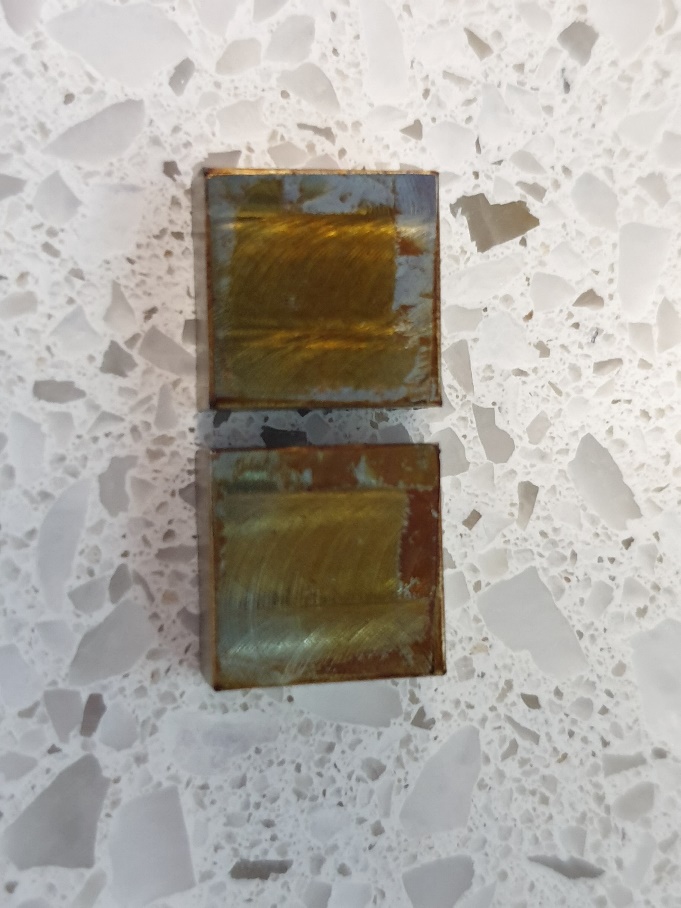


**Figure A.3.** The two films after the bond between them had been broken.

# Antimicrobial Testing of Films by Submersion with and without Washing or Heating

For the antimicrobial testing, 10 μL of m-cresol was pipetted onto 10 mm × 10 mm Si wafers (Ted Pella, Inc.), and samples were made at varying exposures (see Table A.1).

One set of samples was then tested for antimicrobial properties as-prepared (see Fig A.4). Another set of samples were then heated to 210 °C (above the boiling point of m-cresol) for 5 minutes, and washed with distilled water (in which m-cresol is soluble) to remove any possible unreacted m-cresol from the surface (see Fig A.5). *Escherichia coli* K12 (wide type, QUT stock collection) was applied and cultured in an LB medium at 37 ℃ and shaken overnight at 250 rpm till the OD_600_ reached 0.4. Then, 3 mL of the culture was transferred into 50 mL falcon tubes, where the films were submerged for 30 minutes. 100 μL of the culture was then extracted and diluted into a PBS buffer to 1×10^5^ cells/mL. 1 mL of diluted culture was then extracted into a 2 mL Eppendorf tube and 1 μL of SYTOX Green dead cell stain was added into each tube. The SYTOX Green dead cell stain was chosen as it has a high affinity to nucleic acids, and can easily penetrate through the compromised cell membranes to stain the nucleic acids. The dead cell nucleic acid fluoresces bright green upon binding to the SYTOX Green nucleic acid stain when excited at 488 nm ^1^. The tubes were then incubated in a dark room at room temperature for 30 minutes. Flow cytometry was then performed on CytoFlex (BECKMAN COULTER, Life Science, AU). During the flow cytometry, the flowrate of injection was optimized to 35 μL/min and 10,000 cells were counted for analysis of each sample. The signals of forward scatter (FSC), side scatter (SSC), and florescence intensities were detected. The data was then further analyzed by CytExpert software.

**
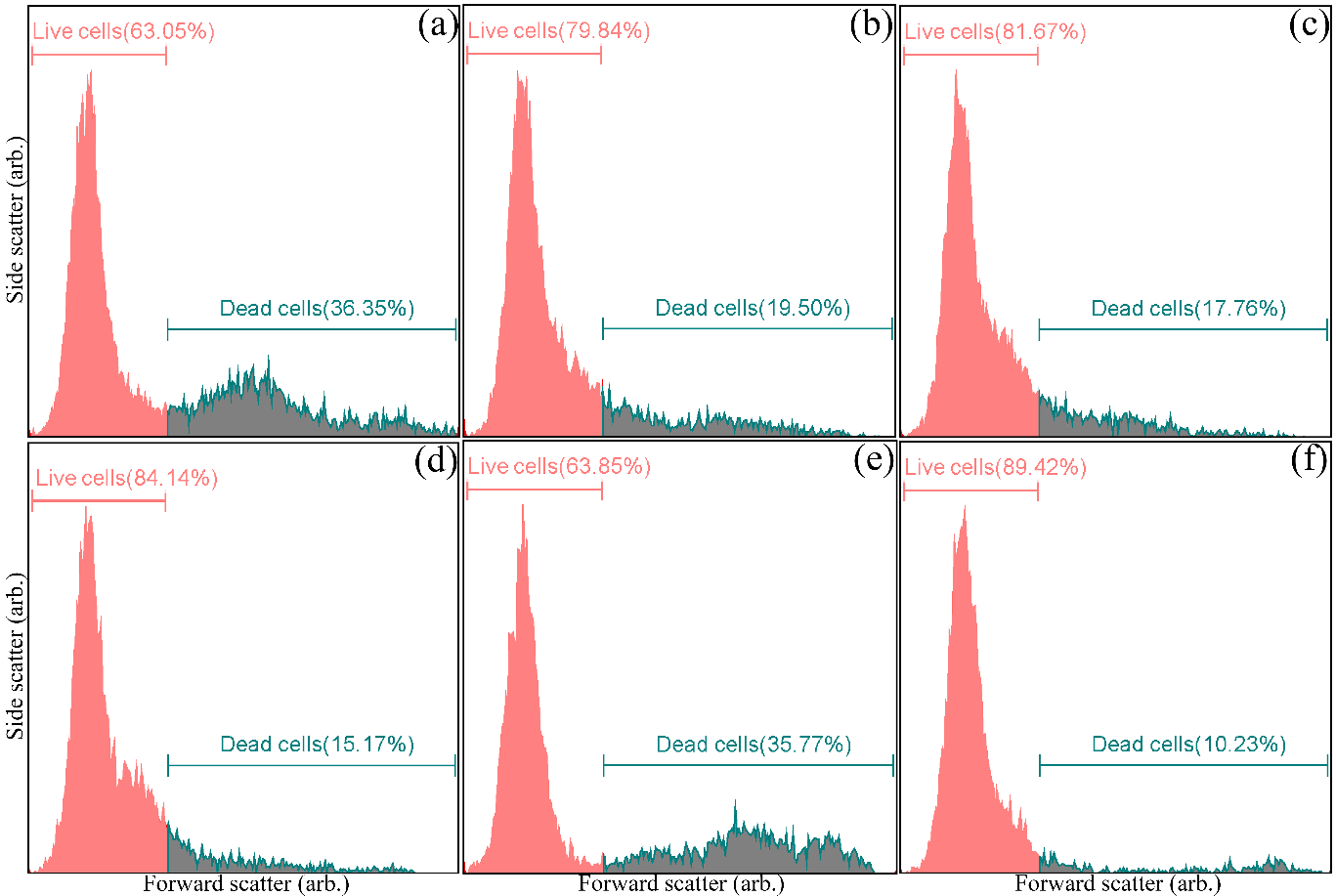
Figure A.4.** The as-prepared samples (a) 0.5 min, (b) 1 min, (c) 2 mins, (d) 4 mins plasma exposures, and (e) 10 μL unreacted m-cresol, (f) control Si wafer, comparing the antimicrobial properties of the coatings at different plasma exposures, on the Si substrate.

**
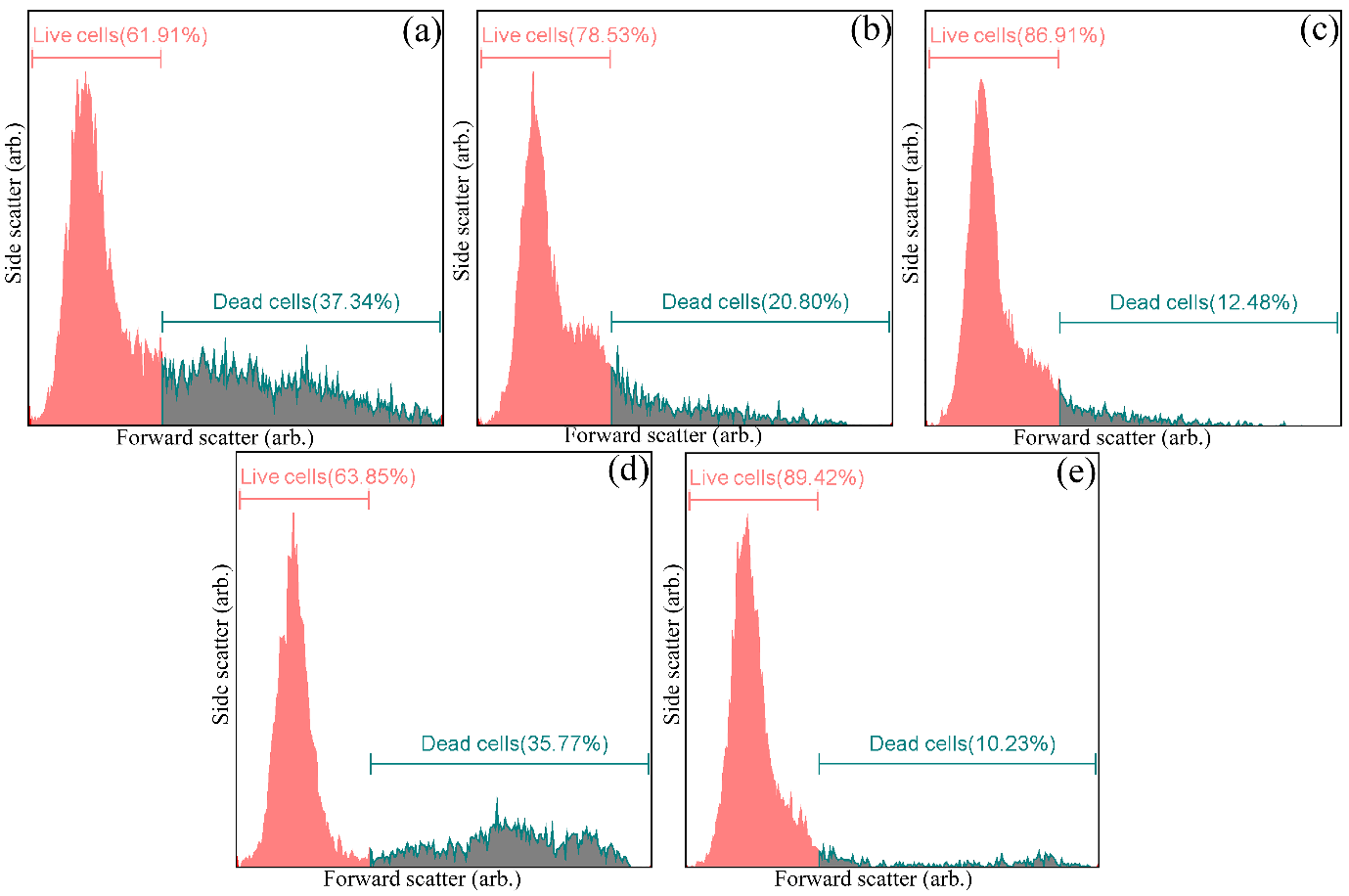
**

**Figure A.5.** The washed and heated samples (a) 0.5 min, (b) 1 min, (c) 2 mins plasma exposures, and (d) 10 μL unreacted m-Cresol, (e) control Si wafer, comparing the antimicrobial properties of the coatings at different plasma exposures, on the Si substrate.

# ToF-SIMS


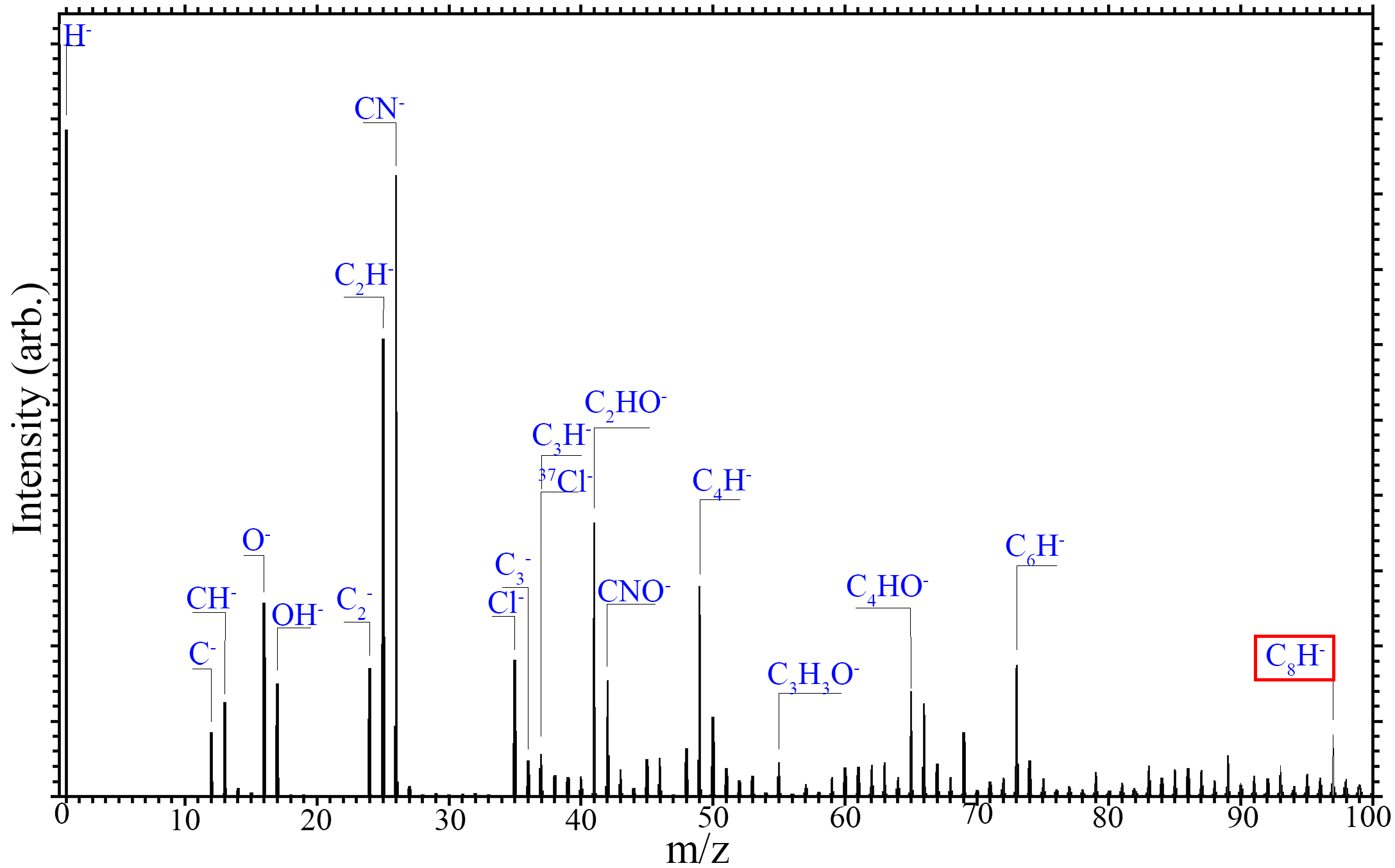


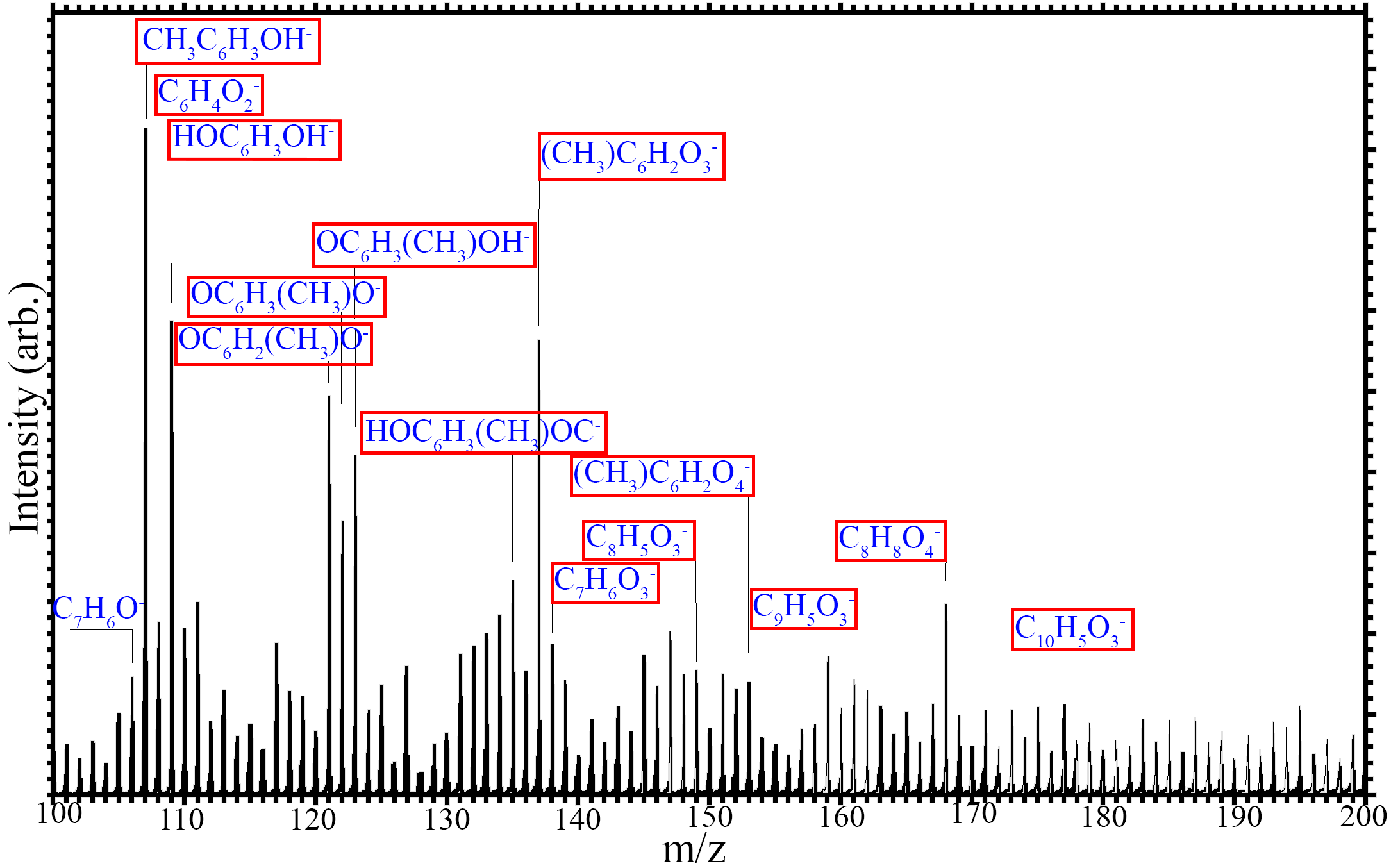


**Figure A.6.** The ToF-SIMS results with negative polarity of an m-cresol film produced with a 1 min plasma exposure, with fragments highlighted in red that suggest oligomerisation or polymerisation of m-cresol.

**
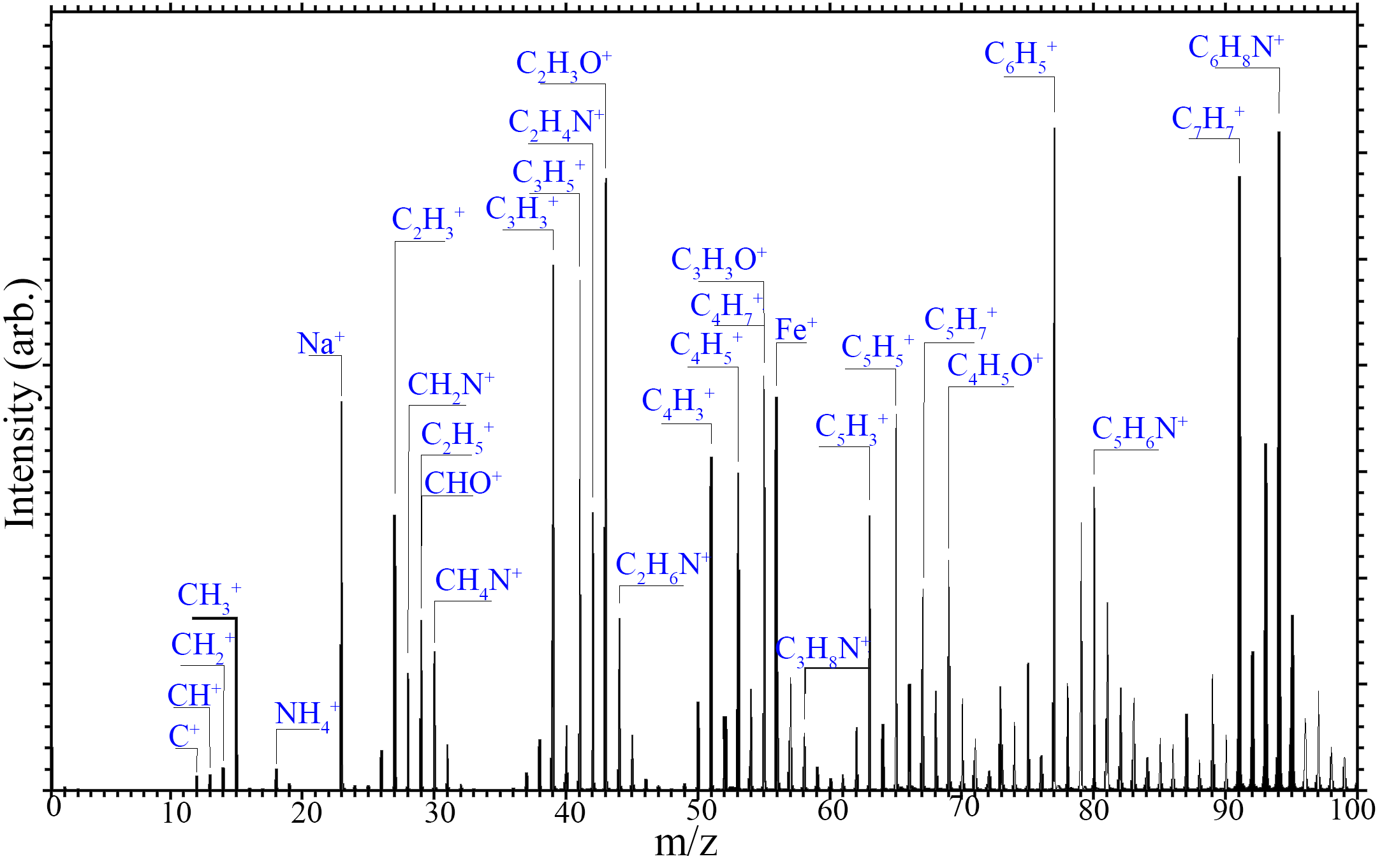
**

**
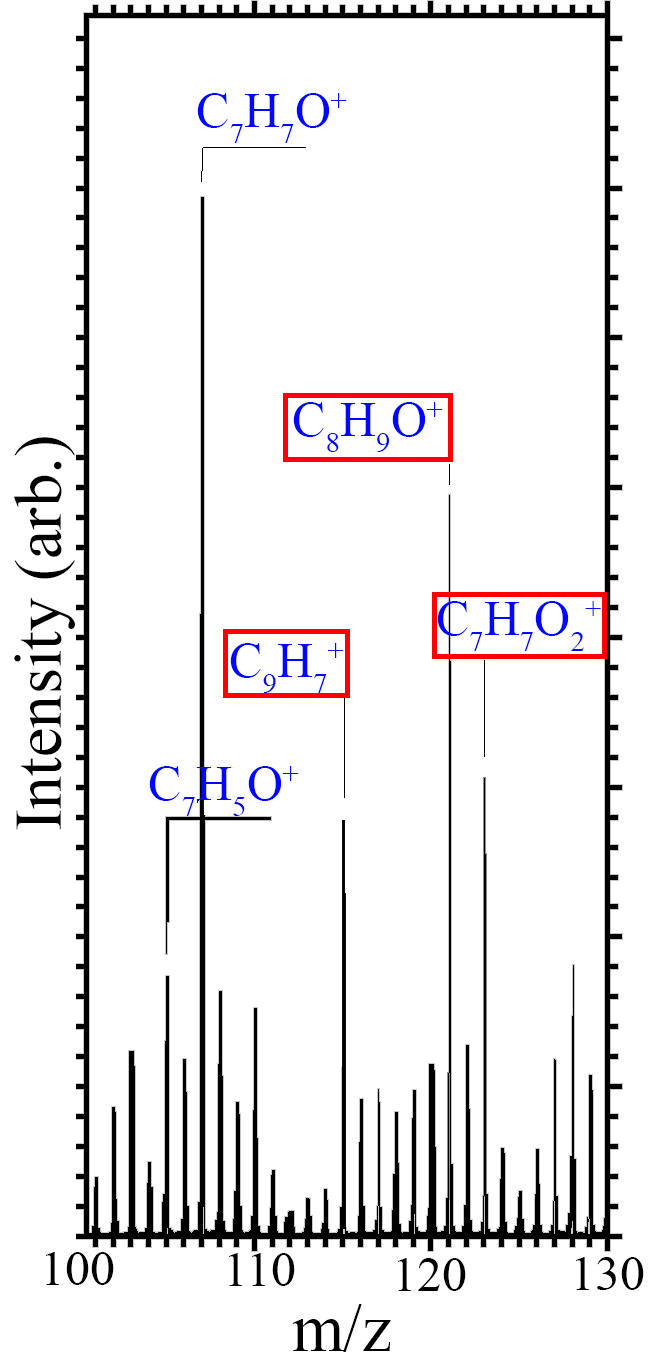
**

**Figure A.7.** The ToF-SIMS results with positive polarity of an m-cresol film produced with a 1 min plasma exposure, with fragments highlighted in red that suggest oligomerisation or polymerisation of m-cresol.

# Spectrometry

UV–vis–NIR spectrophotometry (UV–vis, Cary 5000) was used to investigate the optical properties of the coatings.


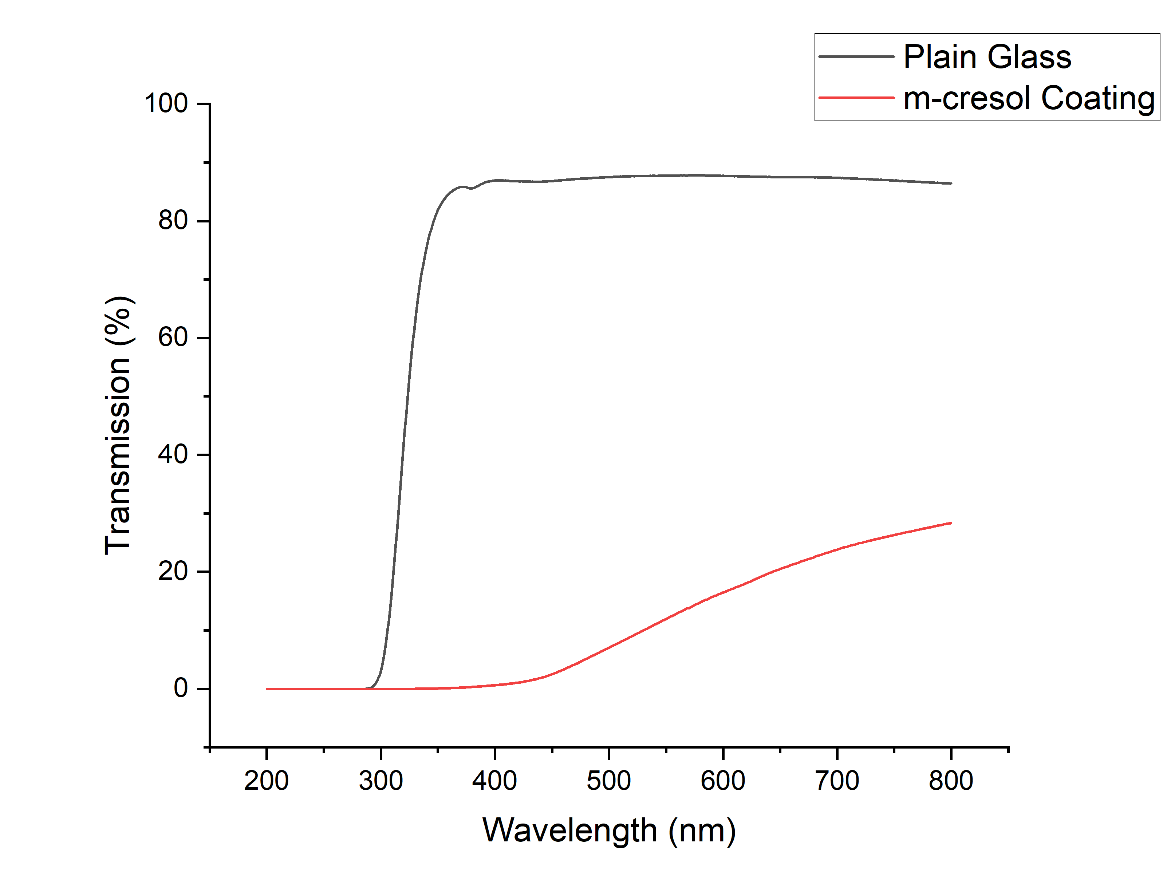


**Figure A.8.** Optical properties of the film produced by a 90 W 1 min plasma exposure on glass, compared to the plain glass.

# Water Contact Angle

A contact angle setup (FTÅ200) was used to measure the contact angle of a water droplet dropped onto the surface of different films at a rate of 2 µL s^−1^.

**Table A.2.** The water contact angle for all samples created.

| Plasma Exposure (mins) | Washed+Heated (°) | As-prepared (°) |
| --- | --- | --- |
| 0.5 | 81.5 ± 5.1 | 67.0 ± 2.2 |
| 1 | 76.9 ± 0.5 | 60.2 ± 1.7 |
| 2 | 83.3 ± 2.4 | 48.8 ± 8.2 |

# Films Made on Other Surfaces


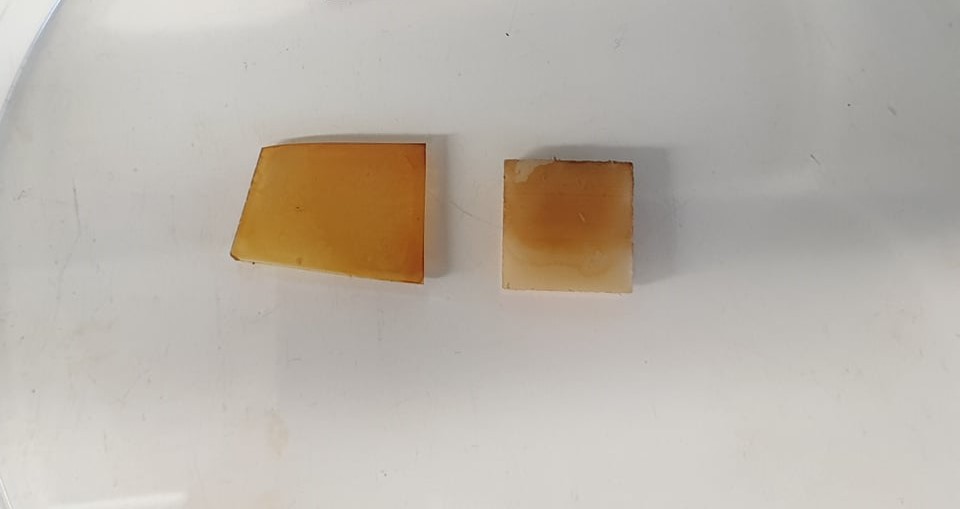


**Figure A.9.** Left, a film made on glass, Right, a film made on Teflon.


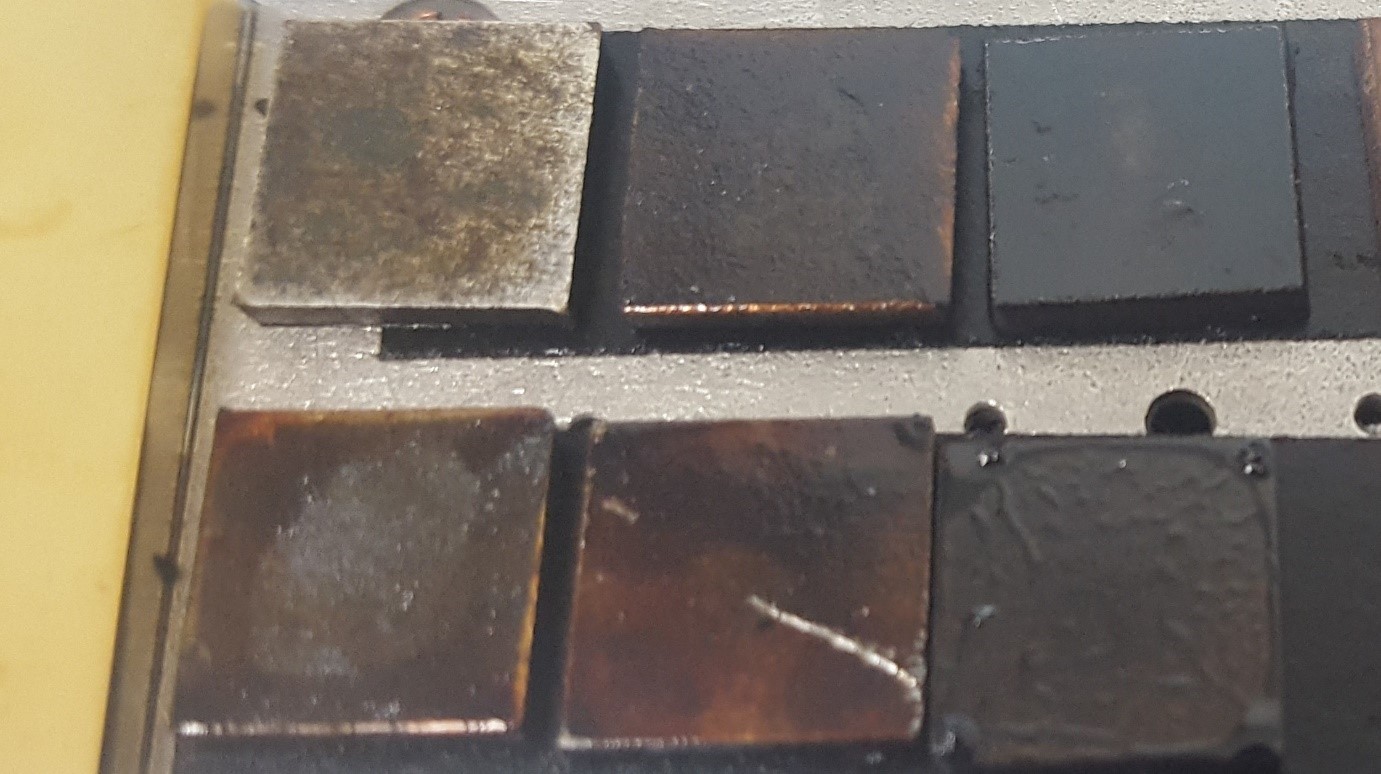


**Figure A.10.** Films made on steel.

# SEM and Optical Microscopy of Films without Washing or Heating


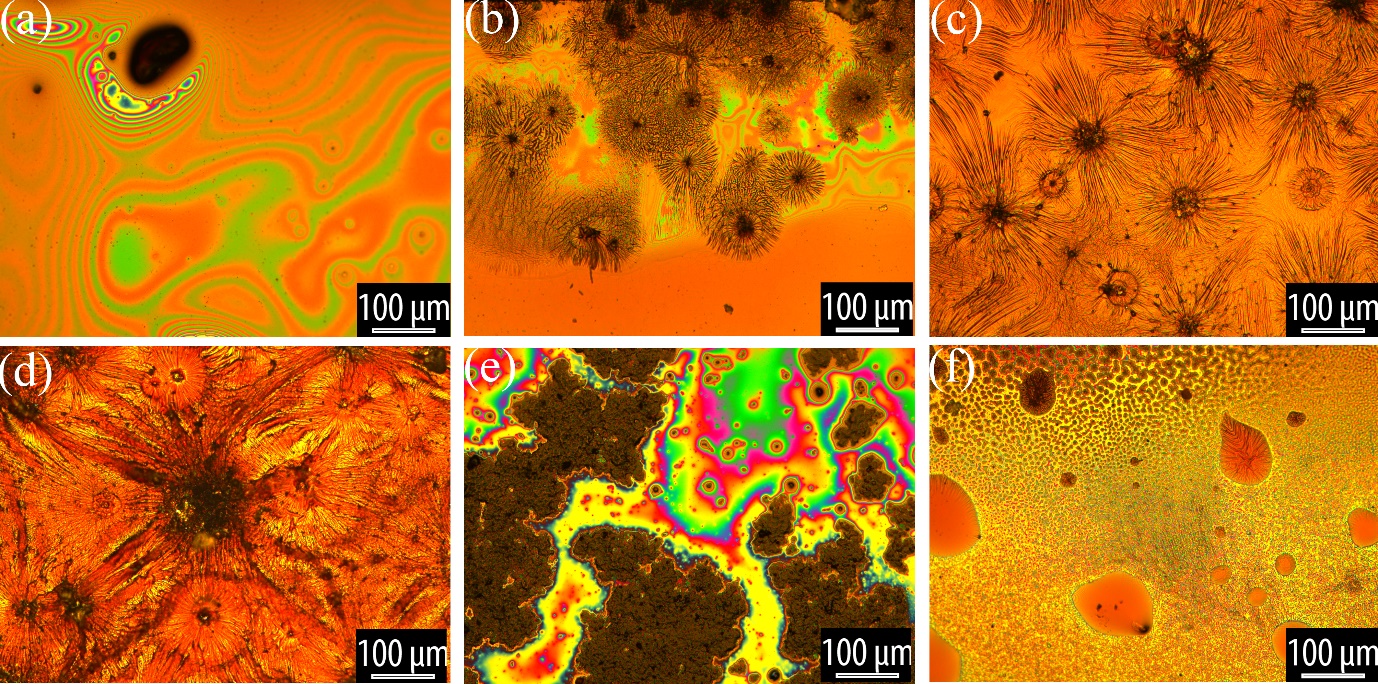
**Figure A.11.** Optical images displaying the features of the films of m-cresol created by plasma exposure durations of (a) 0.5 mins, (b) 1 min, (c) 2 mins, (d) 3 mins, (e) 4 mins, and (f) 5 mins, on the Si substrate.

**
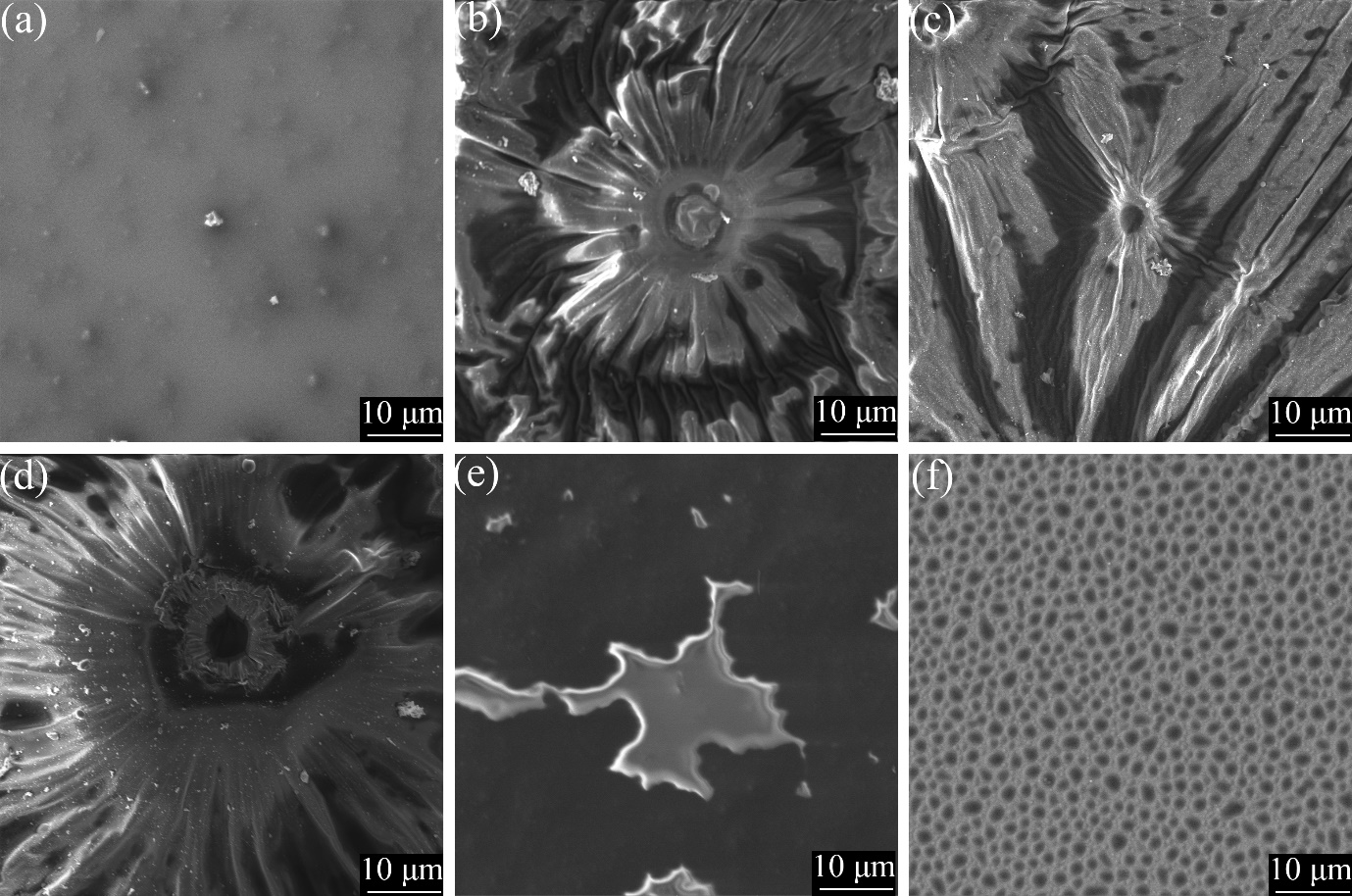
Figure A.12.** SEM images displaying the features of the films of m-cresol created by plasma exposure durations of (a) 0.5 mins, (b) 1 min, (c) 2 mins, (d) 3 mins, (e) 4 mins, and (f) 5 mins, on the Si substrate.

# Adhesion Testing

Samples produced at a plasma exposure duration of 1 min were tested for their adhesion to the substrates by scotch tape lift-off. A length of scotch tape was pressed hard onto each film, and then pulled off. This was performed three times.


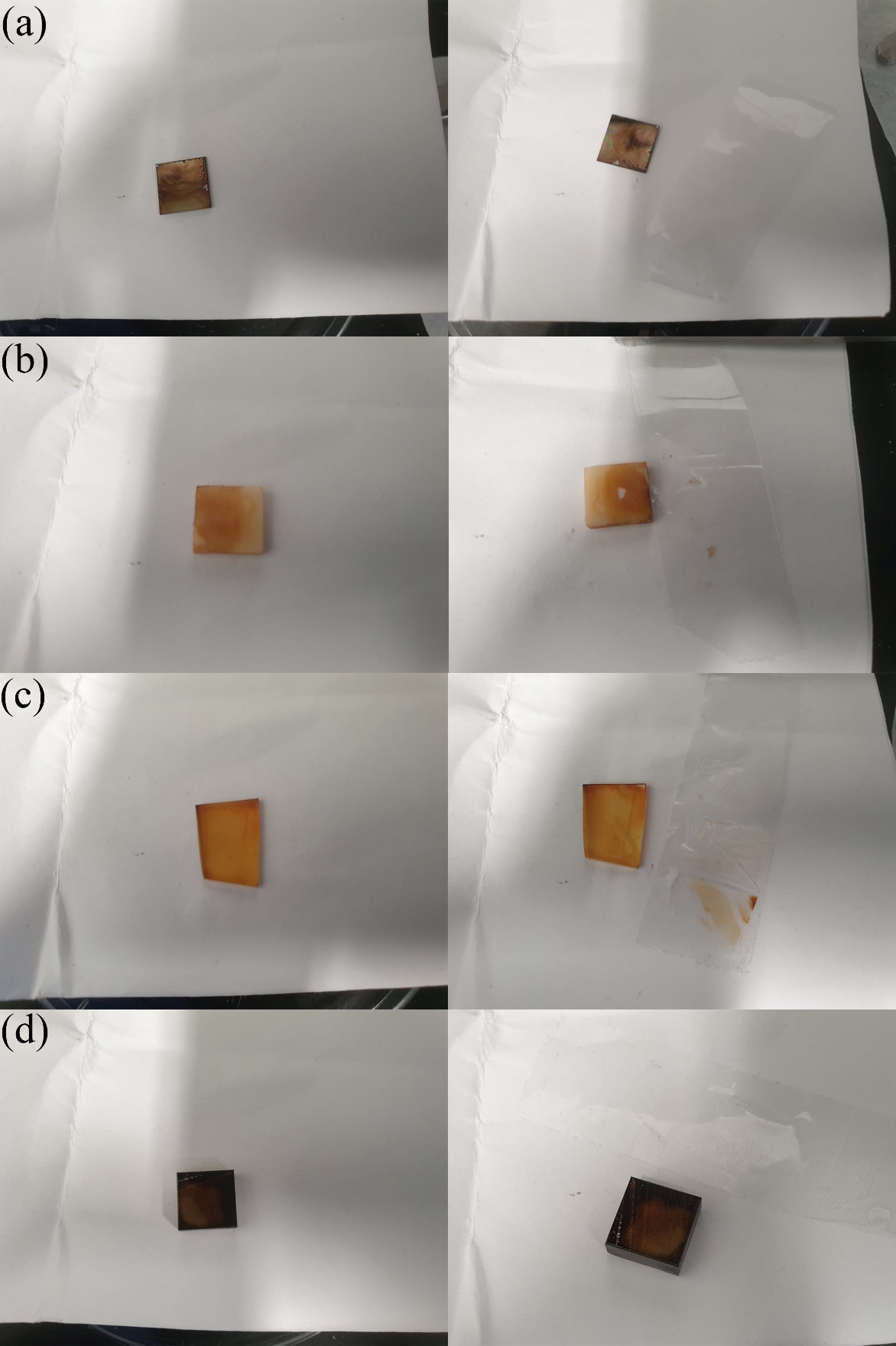


**Figure A.13.** The films on (a) silicon, (b) Teflon, (c) glass, (d) stainless steel. The left images show the films before testing, and the right images after testing.

# Leachable Testing

Leachable testing was performed on the sample produced at 1 min plasma exposure to test if any of the coating leached into water. The coating was produced as per the method in the main text, including the same heating and washing method. 10 μl of distilled water was then pipetted onto the surface of the coating, and left for 15 mins. After 15 mins, the droplet was collected.

The wash from the coating was then compared against known concentrations of the same m-cresol used to produce the coatings by use of Gas Chromatography-Mass Spectrometry (GC-MS, GCMS-TQ8050 NX). The detection limit of the instrument is 2 μg/mL. GC runs were performed over 26mins and m/z of 50-600. The same procedure was also performed on a plain silicon wafer as a control. The results show that the presence of m-cresol is below the 2 μg/mL detection limit of the instrument.


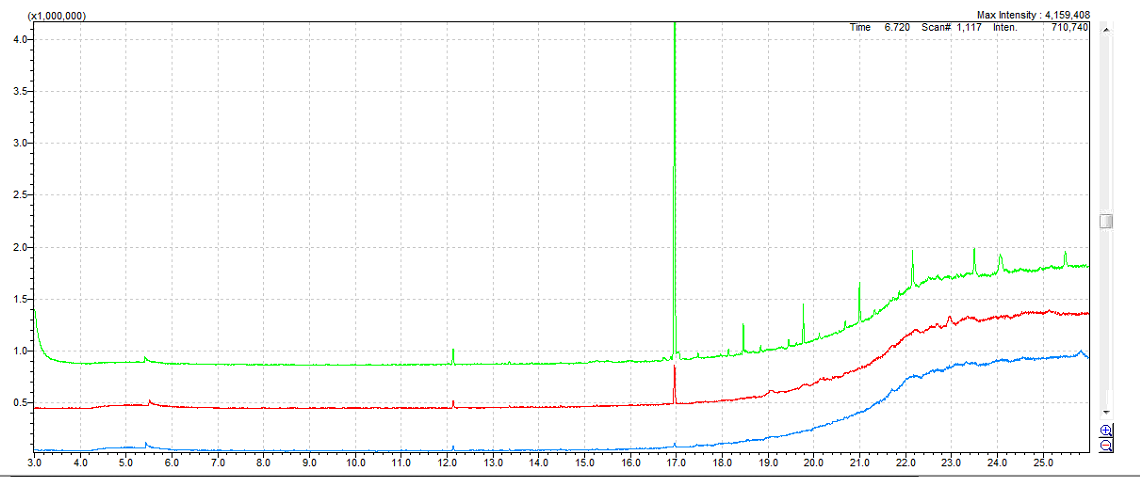


**Figure A.14.** GC-MS of known concentrations of m-cresol. The green trace is at a concentration of 141 μg/mL, the red trace is at a concentration of 14.1 μg/mL, and the blue trace is at a concentration of 1.41 μg/mL. A large peak is seen at the 17 minute mark, for m-cresol.


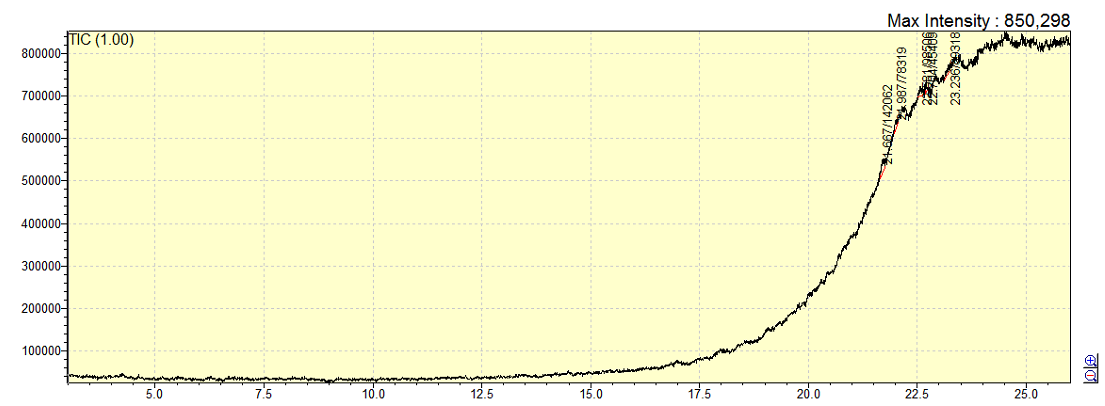


**Figure A.15.** GC-MS testing of wash from the sample produced at a plasma exposure duration of 1 min.


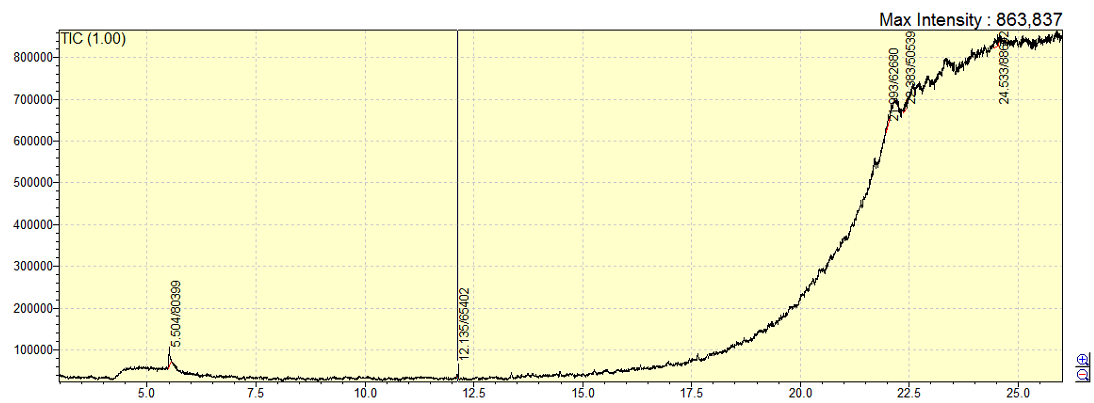


**Figure A.16.** GC-MS testing of wash from silicon wafer control.

The same wash from the coating was then compared against known concentrations of the same m-cresol used to produce the coatings (1/100 dilution in MeOH) by use of Electrospray Ionisation-Mass Spectrometry (ESI-MS, Orbitrap Elite mass spectrometer).

**Figure A.17.** ESI-MS testing of m-cresol standard, with the peak of 107.048 m/z representing m-cresol.

**Figure A.18.** ESI-MS testing of (top) wash from the silicon control, (bottom) wash from the sample produced at a plasma exposure duration of 1 min.

**References**

1. Roth, B. L., Poot, M., Yue, S. T. & Millard, P. J. Bacterial viability and antibiotic susceptibility testing with SYTOX green nucleic acid stain. *Appl. Environ. Microbiol.* **63**, 2421-2431 (1997).
